# Circulating asymmetric dimethylarginine and the risk of preeclampsia: a meta-analysis based on 1338 participants

**DOI:** 10.18632/oncotarget.16543

**Published:** 2017-03-24

**Authors:** Jing Yuan, Xinguo Wang, Yudou Xie, Yuzhi Wang, Lei Dong, Hong Li, Tongyu Zhu

**Affiliations:** ^1^ Department of Medical Information, General Hospital of Jinan Military Command, Shandong 250031, China; ^2^ Department of Medical Information, The Jiaotong Hospital of Shandong Province, Shandong 250031, China; ^3^ Department of Obstetrics and Gynecology, General Hospital of Jinan Military Command, Shandong 250031, China; ^4^ Department of Ultrasonic Medicine, General Hospital of Jinan Military Command, Shandong 250031, China; ^5^ Department of Obstetrics and Gynecology, Center for Reproductive Medicine, Nanfang Hospital, Southern Medical University, Guangzhou 510515, China

**Keywords:** preeclampsia, asymmetric dimethylarginine, endothelial function, meta-analysis

## Abstract

**Background:**

Patients with preeclampsia have higher circulating asymmetric dimethylarginine (ADMA). However, whether circulating ADMA is elevated before the diagnosis of preeclampsia has not been determined.

**Methods:**

A meta-analysis of observational studies that reported circulating ADMA level before the onset of preeclampsia was performed. Pubmed and Embase were searched. Standardized mean differences (SMD) with 95% confidence intervals (CI) were used to estimate the differences in circulating ADMA. A random effect model or a fixed effect model was applied depending on the heterogeneity. The predictive efficacy of circulating ADMA for the incidence of preeclampsia was also explored.

**Results:**

Eleven comparisons with 1338 pregnant women were included. The pooled results showed that the circulating ADMA was significantly higher in women who subsequently developed preeclampsia as compared with those did not (SMD: 0.71, p < 0.001) with a moderate heterogeneity (I^2^ = 43%). Stratified analyses suggested elevation of circulating ADMA is more remarkable in studies with GA of ADMA sampling ≥ 20 weeks (SMD: 0.89, p < 0.01) as compared those with GA of ADMA sampling < 20 weeks (SMD: 0.56, p < 0.01; p for subgroup interaction = 0.03). Differences of maternal age, study design, and ADMA measurement methods did not significantly affect the results. Only two studies evaluated the potential predicting ability of circulating ADMA for subsequent preeclampsia, and retrieved moderate predictive efficacy.

**Conclusions:**

Circulating ADMA is elevated before the development of preeclampsia. Studies are needed to evaluate the predictive efficacy of ADMA for the incidence of preeclampsia.

## INTRODUCTION

Despite of significant improvement of obstetric and perinatal medicines in recent decades, preeclampsia remains one of the major contributors to the maternal and perinatal morbidity and mortality worldwide, particularly in the developing countries [1, 2]. Clinically, preeclampsia is defined as a disorder of pregnancy characterized by hypertension and proteinuria after 20 weeks of gestation in pregnant women with no evidence of previous hypertension [2]. Although preeclampsia has been recognized as a pregnant emergency for a long time, the only cure for it when the manifestation of preeclampsia occurs is delivery [3]. Therefore, early identification of pregnant women who are at higher risk for development of preeclampsia is of important clinical significance since some preventative strategies, such as aspirin has been suggested to be more effective if administered in early pregnancy [4].

Pathophysiologically, preeclampsia is characterized by the impaired trophoblast invasion of the maternal spiral arteries [5]. Consequently, placental hypoxia leads the overproduction and release of placenta derived anti-angiogenic and inflammatory factors, subsequently contributing to the systematic manifestation of preeclampsia [6]. Accumulating evidence from experimental and clinical studies indicates that endothelial dysfunction (ED) is an initial pathophysiological feature of preeclampsia [7]. Therefore, marker of ED may become a predictor for the incidence of preeclampsia. Current understanding of the mechanisms of endothelial function demonstrates that decrease in bioavailability of nitric oxide (NO) is a major contributor to the pathogenesis of ED, and level of asymmetric dimethylarginine (ADMA), an endogenous inhibitor of NO synthase, may be a determinant of ED [8, 9]. Indeed, increased serum ADMA has been associated with many clinical syndromes involving ED, such as hypertension [10], coronary arterial disease [11], stroke [12], and preeclampsia [13]. Interestingly, a previous study summarized case-control studies which evaluated serum ADMA in preeclampsia and showed that pregnant women with preeclampsia have higher serum ADMA as compared with those without preeclampsia [14]. However, studies regarding the serum ADMA level before the clinical diagnosis of preeclampsia yielded different results [14–21]. Therefore, the aim of the current meta-analysis is to systematically evaluate whether circulating ADMA is elevated before the clinical diagnosis of preeclampsia and to explore the predictive efficacy of circulating ADMA for the incidence of preeclampsia.

## RESULTS

### Literature searching results

**Figure 1:**
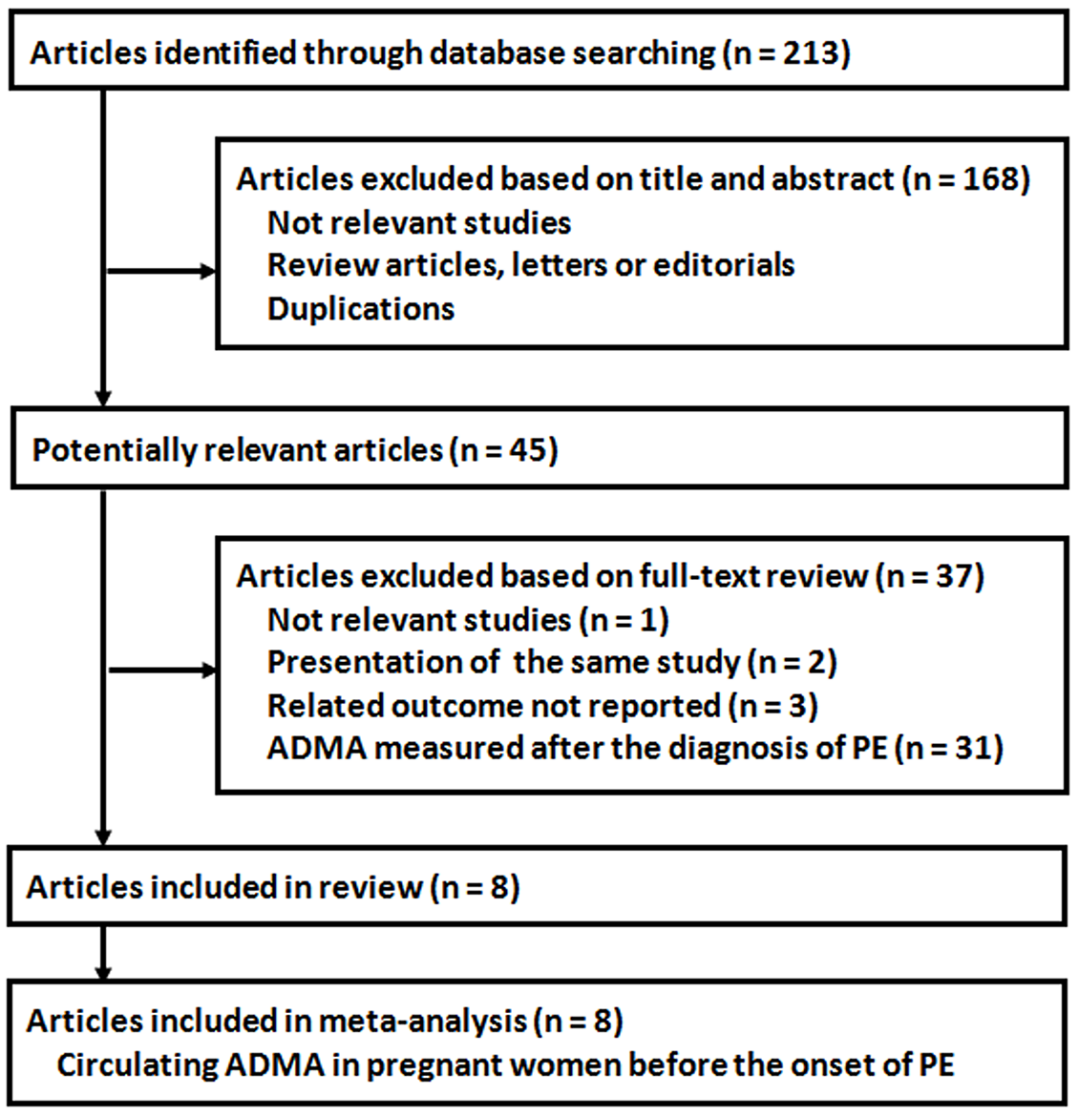
Process of literature searching

The study selection process was shown in Figure 1. Overall, the database searching identified 213 citations from Pubmed and Embase, of which eight published articles were finally included [14–21]. Many of the studies were excluded after full-text review because the circulating ADMA was measured after the diagnosis of preeclampsia. Because three [15, 16, 18] of the include studies reported two sets of circulating ADMA data based on different GA of sampling, eleven comparisons were available for the different of circulating ADMA before the onset of preeclampsia. Only two studies [16, 17] reported data regarding the predictive efficacy of circulating ADMA for the incident risk of preeclampsia.

### Study characteristics

The characteristics of included studies were shown in Table 1. All of the included studies recruited women with apparent normal pregnancies at the enrollment, and seven of them included women with singleton pregnancies only [14, 15, 17–19, 21]. The sample size of the included pregnant women varied from 44 to 375, and the GA of sampling for the measurement of circulating ADMA were from 6 to 25 weeks. The mean maternal age varied from 22.9 to 32.9 years, and the methods for circulating ADMA measuring included high performance liquid chromatography (HPLC), gas chromatography-mass spectrometry (GC-MS) and enzyme-linked immunosorbent assay (ELISA).

**Table 1:** Characteristics of included studies

Author year	Country	Studydesign	No. ofPE	No. ofControl	GA ofsampling	Population	Maternalage	ADMAmeasurement
					wks		years	
Savvidou 2003	UK	Matched case-control	10	43	23~25	Women with singleton pregnancies, on no medications, had no personal or family history of premature cardiovascular disease, and had appropriately grown fetuses for the gestation	28.8	HPLC
Speer 2008	USA	Nested case-control	15	31	9~21	Nulliparous healthy women without known medical complications	22.9	HPLC
Osmanağaoğlu 2011	Turkey	Matched case-control	22	22	6~12	Singleton pregnant women with normal pregnancies	29	ELISA
Rizos 2012a	Greece	Matched case-control	10	41	11~14	Singleton pregnant women with normal pregnancies	32.9	ELISA
Rizos 2012b	Greece	Matched case-control	10	41	20~25	Singleton pregnant women with normal pregnancies	32.9	ELISA
Khalil 2013	UK	Nested case-control	75	300	11~13	Singleton pregnancies delivering phenotypically normal neonates	32.3	GC-MS
Lopez-Alarcon 2015a	Mexico	Cohort	49	179	< 20	Normal blood pressure and singleton pregnancy before 20 weeks of gestation	30.9	HPLC
Lopez-Alarcon 2015b	Mexico	Cohort	49	179	21~24	Normal blood pressure and singleton pregnancy before 20 weeks of gestation	30.9	HPLC
Bian 2015	China	Nested case-control	44	100	12~16	Pregnant women with no systematic diseases	29.1	ELISA
Karampas 2016a	Greece	Matched case-control	12	47	11~13	Singleton pregnant women with normal pregnancies	32.6	ELISA
Karampas 2016b	Greece	Matched case-control	12	47	20~25	Singleton pregnant women with normal pregnancies	32.6	ELISA

Abbreviations: PE, preeclampsia; GA, gestational age; ADMA, asymmetric dimethylarginine; HPLC, high performance liquid chromatography; GC-MS, gas chromatography-mass spectrometry; ELISA, enzyme-linked immunosorbent assay.

### Quality assessment

The overall quality of studies included in the meta-analysis was good, with three studies [15, 18, 21] scoring 9 stars on the Newcastle-Ottawa scale, three studies scoring 8 stars [14, 16, 17] and the other two [19, 20] scoring 7 stars.

### Circulating level of ADMA before the onset of preeclampsia

**Figure 2:**
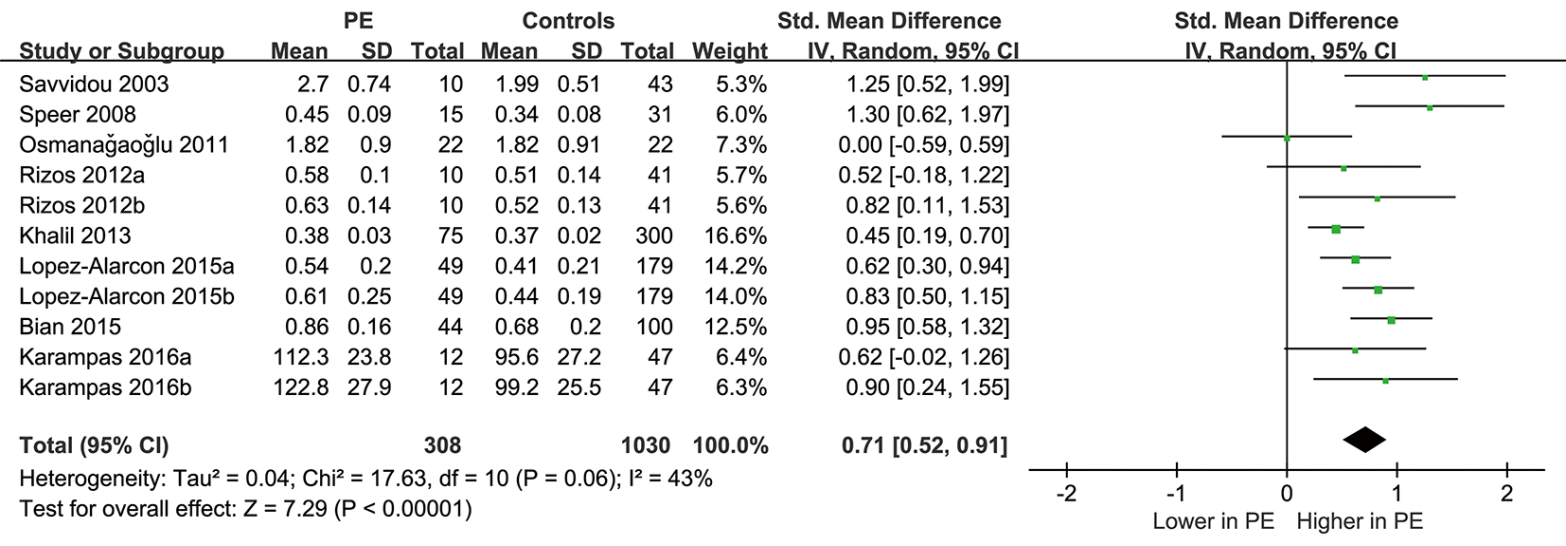
Forest plot for the difference of circulating ADMA before the onset of the disease between women who subsequently developed preeclampsia or not

Overall, eleven comparisons with 1338 pregnant women were included in the meta-analysis, of which 308 women developed preeclampsia during follow-up. The pooled results showed that the circulating level of ADMA was significantly higher in women who subsequently developed preeclampsia as compared with those did not (SMD: 0.71, 95% CI 0.52 to 0.91, p < 0.001; Figure 2), with a moderate heterogeneity among the included studies (Cochrane's Q test, p = 0.06; I^2^ = 43%). Sensitivity analyses by omitting data from one comparison at a time did not significantly change the results, indicating the stability of the pooled outcome (data not shown).

**Figure 3:**
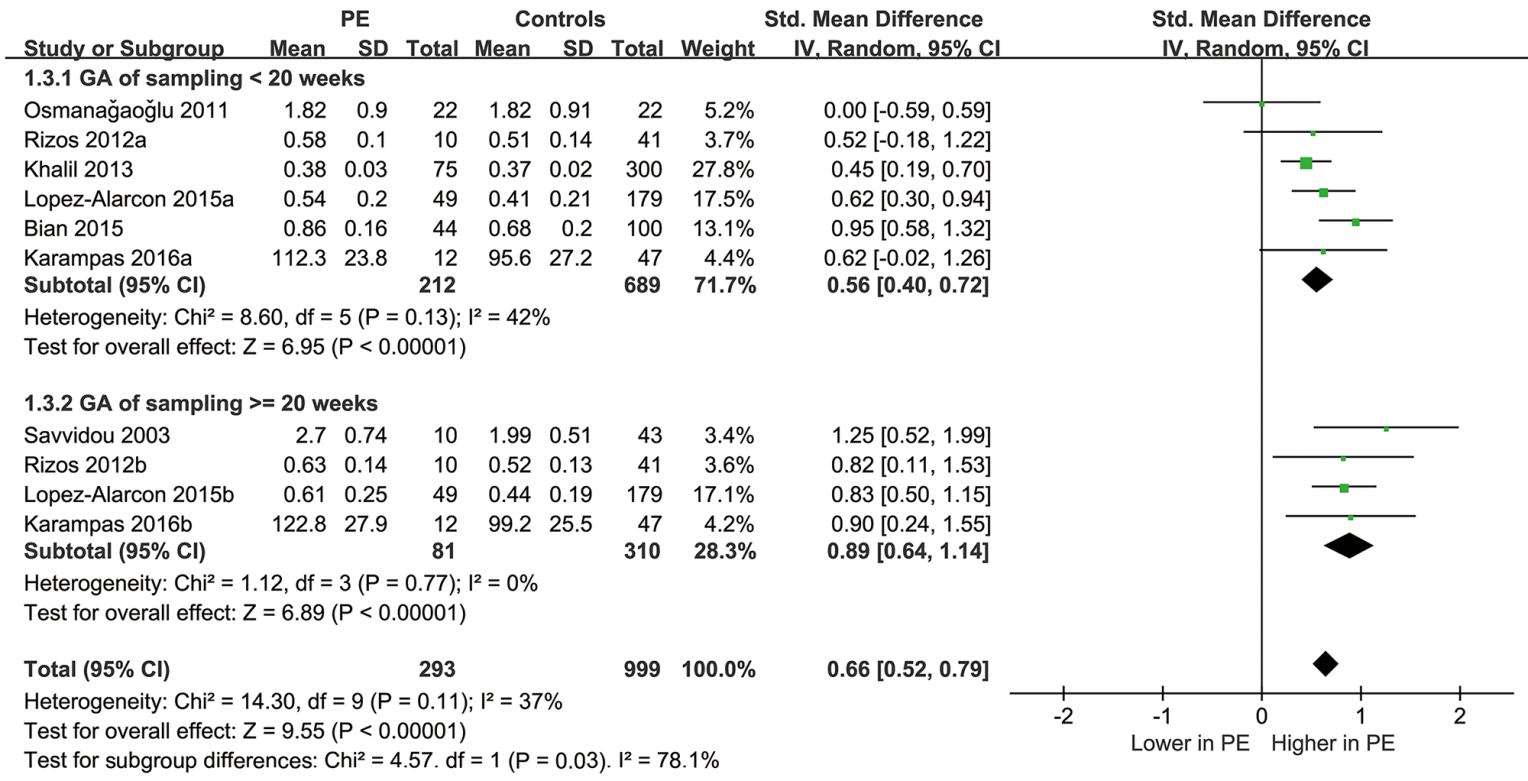
Forest plot for the difference of circulating ADMA before the onset of the disease between women who subsequently developed preeclampsia or not as stratified by the GA of ADMA sampling

Subsequent subgroup analyses indicated that the results consistently showed that circulating level of ADMA was significantly higher in women who subsequently developed preeclampsia in studies of cohort or case-control design, in studies with ADMA sampling at GA of < 20 weeks, ≥ 20 weeks or 9~21 weeks, in studies with maternal age of < or ≥ 30 years, and in studies with ADMA measured by HPLC, ELISA or GC-MS (Table 2). Interestingly, a more remarkably elevated circulating ADMA in women who subsequently developed preeclampsia was noticed in studies with GA of ADMA sampling ≥ 20 weeks (SMD: 0.89, 95% CI 0.64 to 1.14, p < 0.01) compared in studies with GA of ADMA sampling < 20 weeks (SMD: 0.56, 95% CI 0.40 to 0.72, p < 0.01; p for subgroup interaction = 0.03; Figure 3). These results suggested that the GA of ADMA sampling may be a potential determinant of heterogeneity among the included studies, and the elevation of circulating ADMA may be more remarkable after 20 weeks of GA in women who subsequently developed preeclampsia.

**Table 2:** Effects of study characteristics on the ADMA outcome: subgroup analyses

Study characteristics	ADMA				
	Studies (patients), n	I^2^	SMD [95% CI]	p for subgroup effects	p for subgroup interaction
**Study design**					
Cohort	2 (456)	0%	0.73 [0.50, 0.95]	< 0.001	
Case-control	9 (882)	52%	0.72 [0.46, 0.98]	< 0.001	0.99
**GA of ADMA measuring**					
< 20 wks	6 (901)	42%	0.56 [0.33, 0.79]	< 0.001	
≥ 20 wks	4 (391)	0%	0.89 [0.64, 1.14]	< 0.001	0.03^a^
Other (9-21 wks)	1 (46)	—	1.30 [0.62, 1.97]	< 0.001	0.04^b^
**Mean maternal age**					
< 30 years	4 (287)	73%	0.86 [0.31, 1.41]	0.002	
≥ 30 years	7 (1051)	0%	0.62 [0.47, 0.78]	< 0.001	0.42
**ADMA measurement**					
HPLC	2 (456)	0%	0.73 [0.50, 0.95]	< 0.001	
ELISA	3 (565)	76%	0.83 [0.35, 1.30]	< 0.001	
GC-MS	6 (317)	39%	0.66 [0.31, 1.01]	< 0.001	0.85

a, p for interactions between 2 subgroups (< 20 and ≥ 20 wks); b, p for interactions among 3 subgroups.

Abbreviations: GA, gestational age; ADMA, asymmetric dimethylarginine; HPLC, high performance liquid chromatography; GC-MS, gas chromatography-mass spectrometry; ELISA, enzyme-linked immunosorbent assay.

**Figure 4:**
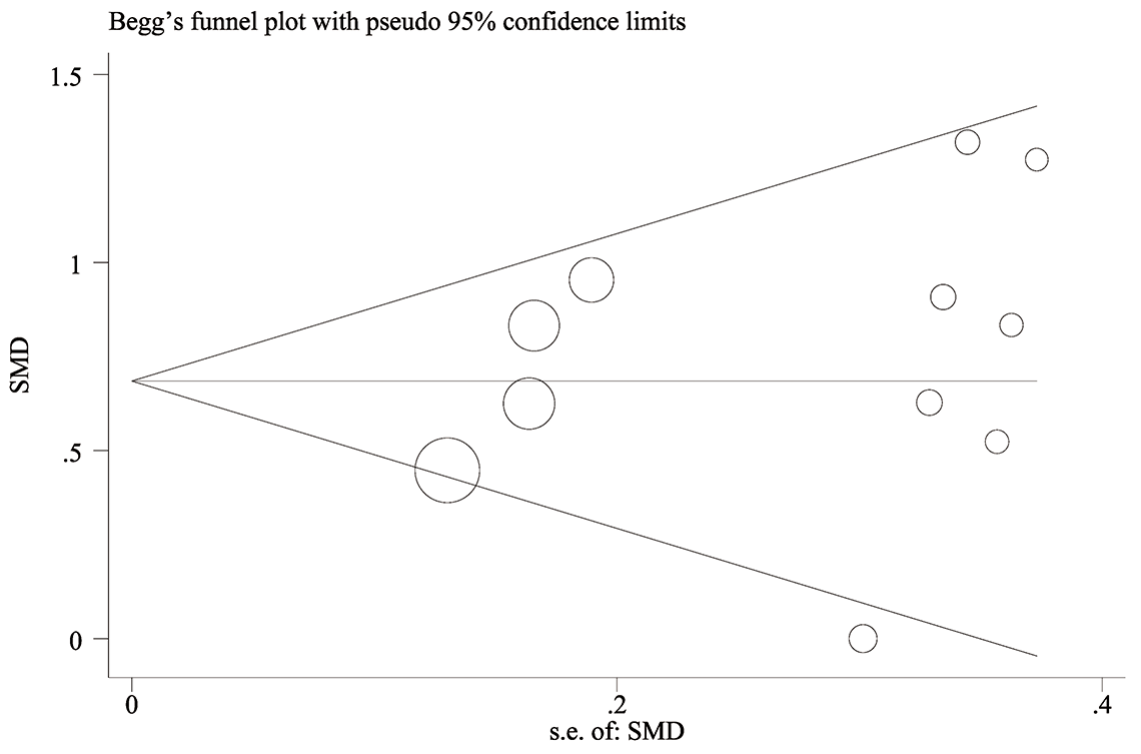
Begg's funnel plot for the evaluation of publication bias

The funnel plot was symmetrical (Figure 4) and the Egger regression test indicated no significant publication bias (Egger test, p = 0.334)

### Predictive efficacy of circulating ADMA for the incidence of preeclampsia

Only two studies [16, 17] provided data on predictive efficacy of circulating ADMA for the incidence of preeclampsia. Because of the limited numbers of available studies, and the potential differences in diagnostic strategies and cutoff ADMA values, a meta-analysis was unable to be performed. Results of the two studies were systematically reviewed. In one study, ADMA was measured at 12~16 weeks of GA, and concluded that the sensitivity, specificity and AUC of the predictive value of ADMA for PE was 0.84, 0.65 and 0.76 respectively [17]. In the other study, ADMA was measured serially, and the increment of ADMA > 80nmol at 1 month to diagnosis of PE was predictive of PE incidence with the sensitivity, specificity and AUC of 0.55, 0.86 and 0.76 [16].

## DISCUSSION

In this meta-analysis, by summarizing all available studies, we found that circulating level of ADMA before the clinical diagnosis of preeclampsia was significantly higher in pregnant women who subsequently developed preeclampsia as compared with those did not. Moreover, results of stratified analyses and sensitivity analyses indicate that these results were not affected by the maternal age, GA of ADMA measuring, study design, and ADMA measurement methods. Interestingly, a more remarkably elevated circulating ADMA in women who subsequently developed preeclampsia was noticed in studies with GA of ADMA sampling ≥ 20 weeks compared in studies with GA of ADMA sampling < 20 weeks. Although elevated circulating ADMA preceding the clinical diagnosis of preeclampsia is confirmed, only two studies evaluated the potential predicting ability of circulating ADMA for subsequent preeclampsia, and retrieved moderate predictive efficacy. Taken together, these results suggest that circulating ADMA is elevated before the disease onset in women who subsequently developed preeclampsia, and elevation of ADMA is more remarkable after 20 weeks of GA. More studies are needed to evaluate the potential predictive efficacy of circulating ADMA for the incidence of preeclampsia.

Preeclampsia is a systematic disease characterized of continuous ED [9]. Previous studies indicated that impairment of NO related vasodilation is the key feature of ED [22]. In vasculatures, NO is generated during the process of L-arginine oxidation via NO synthesize (NOS) [22]. ADMA, as an endogenous inhibitor of NOS, causes ED and participants in the pathogenesis of many vascular diseases, including preeclampsia [9]. Result of our study further confirmed the pathological association between elevated ADMA and subsequent preeclampsia. Moreover, these results also supported by the fact the potential role of L-arginine supplementation, an important mediator of vasodilation, for the prevention of preeclampsia [23].

Results of our study have implications for further studies. Firstly, although previous studies indicates circulating ADMA level is increased in women with preeclampsia, no consensus has been achieved regarding the ADMA level before the onset of the disease. Results of our study confirmed that circulating ADMA is elevated in pregnant women who subsequently developed preeclampsia as early as GA < 20 weeks. The fact that circulating ADMA is elevated preceding the onset of preeclampsia not only supports that elevated ADMA may be involved in the pathogenesis of preeclampsia, but more importantly suggests that measuring of ADMA may be significant for the early identification of pregnant women who are at risk for preeclampsia. This is particularly important considering that limited effective treatments are available if preeclampsia is diagnosed and early application of preventative medication may achieve lower incidence for preeclampsia [24]. Secondly, results of stratified analyses indicate that for pregnant women who subsequently developed PE, more remarkably elevated ADMA is noticed in studies that measuring ADMA at GA > 20 weeks as compared those with GA < 20 weeks. This is consistent with previous notion that ED and elevation of ADMA are early pathophysiological features of preeclampsia which are involved throughout the pathogenesis of preeclampsia [7]. More importantly, the continuously elevated circulating ADMA during the gestational periods of women who subsequently developed preeclampsia ensures the stability of circulating ADMA as a potential marker of preeclampsia. Thirdly, although results of our study support the potential use of circulating ADMA as a marker of preeclampsia risk, only two studies [16, 17] that evaluated the predictive value of ADMA for preeclampsia are available. Reviewing the results of these two studies indicates that the predictive efficacy of circulating ADMA for preeclampsia is moderate, and further studies are needed, particularly focusing on the timing of measurement, the optimal cut-off value, and the potential benefits of serial measurements of ADMA levels.

To the best of our knowledge, our study is the first meta-analysis that confirmed a significant elevation of circulating ADMA before disease onset in women who subsequently developed preeclampsia. However, some limitations of current meta-analysis, mostly inherited from meta-analysis of observational studies, should be considered when interpreting the results. Firstly, moderate heterogeneity was detected among the included studies. Although we performed subgroup analyses to evaluate the potential influence of study characteristics on the study results and found that the GA of ADMA sampling may be a potential determinant of heterogeneity, we could not exclude the existence of other potential factors that may affect the results. Further investigation of the potential source of heterogeneity is prevented by the limited number of studies, and unavailability of individual patient based data. Further studies are needed to determine whether the association between elevated ADMA and preeclampsia risk is more remarkable in certain subgroup of pregnant women. Secondly, results of our study confirmed a temporal association between elevated ADMA and subsequent development of preeclampsia. However, whether elevated ADMA is causative to the development of preeclampsia remains to be determined, optimally via interventional studies which target ADMA lowering. Moreover, since elevated ADMA is associated with many vascular diseases rather than preeclampsia, the association between elevated ADMA and subsequent development of preeclampsia may be confounded by the other clinical factors (medications, dietary factors, comorbidities) which also affect ADMA levels [25]. However, since the studies in our meta-analysis generally included healthy women with normal pregnancies, the chances that other factors may affect the circulating ADMA level are low. At last, only two studies regarding the predictive efficacy of circulating ADMA for the development of preeclampsia are available, and further studies are needed. Indeed, previous studies indicated that the prediction for the risk of preeclampsia may include multiple clinical variables [26, 27]. Whether incorporation of circulating ADMA level to the established model is associated with improved predictive efficacy for the development of preeclampsia deserves further investigation.

In conclusion, circulating ADMA is elevated before the disease onset in women who subsequently developed preeclampsia, and elevation of ADMA is more remarkable after 20 weeks of GA. Studies are needed to evaluate the potential predictive efficacy of circulating ADMA for the incidence of preeclampsia.

## MATERIALS AND METHODS

### Database searching

We followed the instructions of Meta-analysis Of Observational Studies in Epidemiology (MOOSE) guidelines [28] and the Cochrane's Handbook for Systematic Review [29] throughout the design, implementation, analysis, and reporting for this study. The electrical databases of Pubmed and Embase were searched for relevant records, using the terms “asymmetric dimethylarginine” or “ADMA”, paired with preeclampsia, pre-eclampsia, eclampsia, PIH, toxemia, EPH, or “edema-proteinuria-hypertension gestos”. The search was limited to studies in humans and published in English. We also analyzed reference lists of original and review articles using a manual approach. The final literature search was performed on September 25th, 2016.

### Study selection

Studies were included for analysis if they met the following criteria: 1) published as full-length article in English; 2) reported testing of ADMA in serum or plasma of pregnant women with blood sampling before clinical onset of preeclampsia and before 30 weeks of gestation (PE Group); 3) included pregnant women that did not develop preeclampsia during the follow-up as controls (Control Group); 4) described the test results conditional on the occurrence of preeclampsia as means and standard deviations (SDs) in pregnancies before preeclampsia and uncomplicated pregnancies. Because we also would like to summarize the predictive effect of ADMA on the incidence of preeclampsia, studies that described the occurrence of preeclampsia conditional on the test result in such a way that 2 × 2 classification tables could be constructed, or the true- and false-positive values, and true- and false-negative values could be estimated were also included.

Diagnosis of preeclampsia was made in accordance with the criteria of the international Society for the Study of Hypertension in Pregnancy [30], which was defined as persistent high systolic (≥ 140 mmHg) or diastolic (≥ 90 mmHg) blood pressure and proteinuria (≥ 0.3g/24 hours or a dipstick result of ≥ 1+, equivalent to 30 mg/dl in a single urine sample or spot urine protein/creatinine ratio ≥ 30 mg protein/mmol creatinine) of new onset after 20 weeks of gestation. Reviews, editorials, abstracts, or duplications of the same studies were excluded.

### Data extraction and quality assessment

Two authors performed the literature searching, data extraction, and quality assessment independently according to the inclusion criteria. Discrepancies were resolved by consensus. The primary outcome of the current study was the difference of circulating ADMA level between the PE and Control Groups before the onset of preeclampsia. The secondary outcomes were the predictive efficacy of circulating ADMA level of the incidence of preeclampsia as summarized by sensitivity, specificity, and the area under the receiver operating characteristic (AUC) curve. For each included study, data on clinical characteristics of the women (age, obstetric history), characteristics of the study design (cohort or case-control), characteristics of ADMA assessing methods, and data related to the interested outcomes were extracted independently by two independent reviewers using standardized data extraction forms. The quality of each study was assessed using the Newcastle-Ottawa Scale [31]. This scale varies from 1 to 9 stars and judges each study on three broad categories: selection of the study groups; the comparability of the groups; and the ascertainment of the outcome of interest.

### Statistics

For the studies with ADMA concentration reported as a continuous variable, we assessed the differences in circulating ADMA between women who did (PEs) and did not develop preeclampsia (Controls) and expressed the results in standardized mean differences (SMD) with 95% confidence intervals (CI). Inter-study heterogeneity was formally tested using Cochrane's test, and significant heterogeneity was considered existing if p value was < 0.10 [32]. The I^2^ statistic was also examined, and a value of I^2^ > 50% indicated significant heterogeneity among the trials [33]. A random-effect model was applied to combine the data if significant heterogeneity was detected; otherwise, a fixed-effected model was used. Sensitivity analysis by omitting one study a time was performed to evaluate the stability of the outcome [29], and subgroup analyses according to the different study design, ADMA measurement methods, gestational age (GA) of ADMA measuring, and maternal age at sampling were performed to evaluate the potential influence of above variables on outcomes. The medians of continuous variables were chosen as the cutoff values for stratification. For studies that reported the predictive efficacy of circulating ADMA on incidence of preeclampsia, sensitivity, specificity and AUC were summarized. Potential publication bias was assessed with Egger regression asymmetry test and funnel plots [34]; p values were two-tailed and statistical significance was set at 0.05. Meta-analysis and statistical analysis was performed with RevMan software (Version 5.3; Cochrane Collaboration, Oxford, UK) and Stata software (version 12.0; Stata Corporation, College Station, TX, USA).
